# Enhancing Fatty Acid Catabolism of Macrophages Within Aberrant Breast Cancer Tumor Microenvironment Can Re-establish Antitumor Function

**DOI:** 10.3389/fcell.2021.665869

**Published:** 2021-04-15

**Authors:** Yucui Gu, Xingjian Niu, Lei Yin, Yiran Wang, Yue Yang, Xudong Yang, Qingyuan Zhang, Hongfei Ji

**Affiliations:** ^1^Department of Medical Oncology, Harbin Medical University Cancer Hospital, Harbin Medical University, Harbin, China; ^2^Institute of Cancer Prevention and Treatment, Harbin Medical University, Harbin, China; ^3^Heilongjiang Academy of Medical Sciences, Harbin, China

**Keywords:** triple-negative breast cancer, tumor metabolic microenvironment, macrophages, fatty acid catabolism, peroxisome proliferator-activated receptor alpha

## Abstract

Triple-negative breast cancer (TNBC) remains an intractable challenge owing to its aggressive nature and lack of any known therapeutic targets. Macrophages play a crucial role in cancer promotion and poor prognosis within the tumor microenvironment (TME). The phagocytosis checkpoint in macrophages has broader implications for current cancer immunotherapeutic strategies. Here, we demonstrate the modulation in the antitumor activity of macrophages within the aberrant metabolic microenvironment of TNBC by metabolic intervention. The co-culture of macrophages with TNBC cell lines led to a decrease in both their phagocytic function and expression of interleukin (IL)-1β and inducible nitric oxide synthase (iNOS). The transcription of glycolysis and fatty acid (FA) catabolism-related factors was inhibited within the dysregulated tumor metabolic microenvironment. Enhancement of FA catabolism by treatment with the peroxisome proliferator-activated receptor-alpha (PPAR-α) agonist, fenofibrate (FF), could re-establish macrophages to gain their antineoplastic activity by activating the signal transducer and activator of transcription 1 (STAT1) signaling pathway and increasing ATP production by FA oxidation. The combination of fenofibrate and anti-CD47 therapy significantly inhibited tumor growth in a 4T1 tumor-bearing mouse model. In conclusion, the enhancement of FA catabolism of macrophages could re-establish them to resume antitumor activity in the TME. Anti-CD47 therapy combined with fenofibrate may serve as a novel and potential immunotherapeutic approach for the treatment of TNBC.

## Introduction

Breast cancer is one of the most common forms of cancer and the second most common cause of mortality among women worldwide (Wahba and El-Hadaad, 2015). Triple-negative breast cancer (TNBC) accounts for 15–20% of all newly diagnosed breast cancers (Anders and Carey, 2008) and is a clinical challenge, owing to the lack of any effective targeted therapy (Lehmann et al., 2011; Metzger-Filho et al., 2012). Immunotherapy with immune checkpoint blockers has demonstrated clinical efficacy and survival benefit in advanced TNBC (Schmid et al., 2018), but its success was observed only in a minority of patients. Therefore, enhancing the efficacy of immunotherapy has become the focus of future research.

Tumor development and progression are influenced by the crosstalk between tumor cells and their microenvironments. The tumor microenvironment (TME) is a complex structure composed of multiple cell types aside from the tumor cells, including myeloid cells, represented by different populations of macrophages. Macrophages can play different roles depending on their distinct phenotypes (Ohri et al., 2009; Ch’ng et al., 2013; Pinto et al., 2019). The antitumor function of macrophages is largely regulated by tumor environmental signals, including chemokines and cytokines, as well as metabolism signals (Murray et al., 2014; Ostuni et al., 2015; Varol et al., 2015). However, the effects of chemokines and cytokines may be insufficient to alter the function of macrophages, especially in the absence of adequate macromolecule supply of building blocks and ATP for energy (Penkert et al., 2016).

The aberrant energy metabolisms of tumor cells and their surrounding stromal environment have become one of the new “hallmarks of cancer” (Hanahan and Weinberg, 2011). Nutrients are sufficient during early tumor formation but may become inadequate with tumor growth. Macrophages often encounter deprivation of some nutrients, including glucose, because of the rapid proliferation of tumor cells (Hamanaka and Chandel, 2012; Dehne et al., 2017). The intrinsic signal of macrophages regulated by the challenging metabolic microenvironment needs to be further determined. In this study, we investigated the effect of metabolic stress on macrophages in the TME and further re-established their antitumor function by metabolic intervention.

## Materials and Methods

### Reagents and Cell Lines

Lipopolysaccharide (LPS) (HY-D1056), phorbol-12-myristate-13-acetate (PMA) (HY-18739), fenofibrate (FF) (HY-17356), and recombinant human interferon (IFN)-γ (HY-P7025) were purchased from MedChemExpress (New Jersey, United States). Anti-CD47 mAb clone MIAP410 (BE0283) was purchased from Bio X Cell (New Hampshire, United States). Filgotinib (M2346) was supplied by AbMole (Houston, United States), and SRT1720 (A4180) was purchased from APExBIO (Houston, United States).

The TNBC cell lines MDA-MB-231 and MDA-MB-468, the human monocyte cell line THP-1, and the mouse 4T1 cell line were purchased from the Type Culture Collection of the Chinese Academy of Sciences (Shanghai, China). All cell lines were authenticated under short tandem repeat (STR) analysis. The cells were preserved in liquid nitrogen and passaged for less than 6 months in our laboratory and all experiments were performed with mycoplasma-free cells.

### Cell Culture and Sample Preparation

MDA-MB-231, MDA-MB-468, and THP-1 cell lines were maintained in Roswell Park Memorial Institute (RPMI)-1640 medium supplemented with 10% fetal bovine serum. THP-1 cells were incubated with 320 nm PMA for 24 h and treated with LPS (100 ng/mL) and IFN-γ (1 μg/mL) for another 24 h. The cells were then incubated with either the normal RPMI-1640 medium (CTRL) or RPMI-1640 low-glucose medium with 0.1 mM palmitic acid and 1 mM oleic acid (Con-CM) for 72 h. MDA-MB-231 or MDA-MB-468 cells were cultured to approximately 70% confluency and then co-cultured with macrophages that had completed induction with LPS and IFN-γ for 72 h (Co-CM1, Co-CM2). For drug treatment experiments, tumor cells were removed and 50 μM FF was added to co-cultured media (Co-CM + FF) for 24 h after the co-culture of macrophages with tumor cells or the conditioned media (Con-CM + FF) for 48 h. Finally, the tumor cells were incubated in the collected supernatant of cultured macrophages in each group, which was added with 20% fresh medium.

### Immunofluorescence Staining

Approximately 3 × 10^5^ macrophage cells cultured in 24-well plates were fixed with 4% paraformaldehyde at room temperature for 30 min, permeabilized with 0.5% Triton X-100 for 30 min, and blocked with 1% bovine serum albumin for 1 h. The cells were incubated with anti-IL-1β (BM0962, 1:200; Boster, Wuhan, China), anti-iNOS (BM0362, 1:200; Boster, Wuhan, China), or anti-CD68 (ab125047, 1:200;Abcam, Cambridge, MA, United States) antibodys at 4°C overnight. The next day, the macrophages were incubated with Alexa Fluor 488- and 594-conjugated secondary antibodies (1:200; Thermo Fisher Scientific, Carlsbad, United States) at room temperature for 1 h. Following three washes with phosphate-buffered saline (PBS), cells were mounted with medium containing 4′,6-diamidino-2-phenylindole (DAPI) (MedChemExpress, New Jersey, United States) and the samples were imaged with an Axioskop 2 mot plus fluorescence microscope equipped with Plan Apochromat 203/0.8 NA and 403/0.95 NA objectives and an AxioCam MRc camera with AxioVision software 4.7.1 (Carl Zeiss AG, Jena, Germany).

### Ink Phagocytosis Assay

A total of 1.6 × 10^5^ macrophage cells were spread on the bottom of a 6-well plate. The ink was diluted five times and added to each well at a volume of 40 μL. The plates were incubated at 37°C for approximately 3 h. Once the ink particles swallowed by macrophages were visible, the cells were washed with serum-free RPMI-1640 medium for three times, fixed with methanol at room temperature for 1 h, stained with eosin for 1 min, sealed with neutral balsam and observed under a microscope and photographed. The phagocytic rate was calculated by counting 200 cells.

### Real-Time Quantitative Polymerase Chain Reaction

Total RNA from macrophages was extracted with Trizol reagent (Thermo Fisher Scientific, Carlsbad, United States) and 500 ng of RNA was reverse transcribed to cDNA using a Transcriptor First Strand cDNA Synthesis Kit (Roche, Switzerland, Germany). Real-time quantitative polymerase chain reaction (RT-PCR) was performed using the SYBR Green I real-time detection kit (CWBio, Beijing, China) on a CFX96 Detection System (Bio-Rad, California, United States). The results were normalized to the internal standard mRNA levels using the 2^–ΔΔ^ ^CT^ method. Primer sequences are listed in Table 1.

**TABLE 1:** Primers used for the analysis of transcript levels of factors in macrophages.

Gene name (Forward-F and Reverse-R)	Primer sequences
IL-1β-F	CTCTTGTTGATGTGCTGCTG
IL-1β-R	GACCTGTTCTTTGAAGTTGACG
iNOS-F	CTCTACAACATCCTGGAGCAAGTG
iNOS-R	ACTATGGAGCACAGCCACATTGA
GLUT1-F	TGTGGGAGGAGCAGTGCTCG
GLUT1-R	TGGGCTCTCCGTAGCGGTG
HK2-F	TGATCGCCTGCTTATTCACGG
HK2-R	ACCGCCTAGAAATCTCCAGAAGG
PPARα-F	AGAGCCCCATCT GTCCTCTC
PPARα-R	ACTGGTAGTCTG CAAAACCAAA
SLC27A4-F	TGAGTTTGTGGGTCTGTGGCTAGG
SLC27A4-R	AAGACAGTGGCGCAGGGCATC
CPT1a-F	GGGATCGATCGTCACCTCTTC
CPT1a-R	GTCAAACAGCTCCACTTGCTG
ACADM-F	AAGCAGGAGCCCGGATTAGG
ACADM-R	TCCCCGCTTTTGTCATATTCC
si-PPARα-F	GGAUAGUUCUGGAAGCUUUTT
si-PPARα-R	AAAGCUUCCAG AACUAUCCTC

### Western Blot Analysis

Cells were lysed in radioimmunoprecipitation assay (RIPA) buffer, and proteins were denatured and separated by electrophoresis. Proteins were electro-transferred onto polyvinylidene fluoride membranes according to the manufacturer’s instructions (Invitrogen). The membranes were incubated overnight at 4°C with IL-1β (1:400), iNOS (1:1000), IL-10 (BA4317-2, 1:1000; Boster, Wuhan, China), PPAR-α (ab215270, 1:1,000; Abcam, Cambridge, MA, United States), CPT1A (ab220789, 1:1,000; Abcam, Cambridge, MA, United States), JAK1 (ab133666, 1:5,000; Abcam, Cambridge, MA, United States), STAT1 (ab109320, 1:10,000; Abcam, Cambridge, MA, United States), and SIRT1 (ab189494, 1:1,000; Abcam, Cambridge, MA, United States) antibodies and further probed with anti-mouse IgG (1:3,000, Cell Signaling, Boston, United States) and anti-rabbit IgG (1:3,000, Cell Signaling, Boston, United States). Protein expression was visualized by enhanced chemiluminescence (ECL; Promega, Madison, WI, United States).

### Colony-Formation Assay

The tumor cells were seeded in 60 mm Petri dishes at a density of 1 × 10^3^ cells/well and cultured in conditioned supernatant (from Co-CM1,Co-CM1 + FF,Con-CM,Con-CM + FF,respectively), which was added with 20% fresh medium, and replaced every 2 days. The culture was terminated when visible clones appeared on the petri dish. The clones were fixed with 4% paraformaldehyde and then stained with 0.1% crystal violet for visualization 12 days later.

### Enzyme-Linked Immunosorbent Assay

After macrophage cells were stimulated under different conditions (Co-CM1, Co-CM1 + FF, Con-CM, Con-CM + FF groups), the supernatant was collected and centrifuged at 1,800 × *g* at 4°C for 10 min. IL-12 and IL-23 levels were measured using IL-12 (ab46037, Abcam) and IL-23 (KIT034, Baiye Biotechnology, Shanghai, China) enzyme-linked immunosorbent assay (ELISA) kits in accordance with the manufacture’s instructions. Each experiment was repeated three times. The final results were pooled as the mean concentration of cytokines.

### Non-Esterified Free Fatty Acids Assay

Non-esterified free fatty acids (NEFA) were measured using a commercially available assay according to the manufacturer s instructions (BC0595, Beijing Solarbio). A standard curve was made from 0.05 to 1 μmol/mL serial dilution in a 96-well plate, then followed by adding 10 μL of each group sample, finally addition of the manufacturer s reagents, and the absorbance value was measured at 550 nm.

### Oxygen Consumption Rate and Extracellular Acidification Rate Assay

The extracellular acidification rate (ECAR) and cellular oxygen consumption rate (OCR) were measured using Seahorse XF 96 Extracellular Flux Analyzer (Seahorse Bioscience). Macrophages cells from CTRL and Co-CM1 groups (1 × 10^4^ cells/well) were seeded into a Seahorse XF 96 cell culture microplate. For the ECAR assay, after baseline measurements, D-glucose (10 mmol/L), oligomycin (1 μmol/L), and 2-deoxyglucose (50 mmol/L) were sequentially injected into each well. For the OCR assay, oligomycin (1 μmol/L), carbonyl cyanide 4-(trifluoromethoxy) phenylhydrazone (FCCP, 0.5 μmol/L), and rotenone/antimycin A (1 μmol/L) were sequentially injected. Data were assessed using the Seahorse XF-96 software.

### Evaluation of Tumor-Infiltrating Immune Cells and Immunohistochemistry (IHC)

Gene expression data were used to infer the relative proportions of 22 types of infiltrating immune cells using the CIBERSORT algorithm (Chen et al., 2018). TNBC samples from 104 patients without neoadjuvant therapy were obtained for this retrospective study from May 2006 to December 2014. The slides of humans were incubated with antibodies against the macrophage marker CD68 (1:200 dilution), and M1 macrophage marker CD86 (ab239075, 1:200 dilution; Abcam, Cambridge, MA, United States). Antibodies against PPAR-α (1:200 dilution), iNOS (1:200 dilution), and F4/80 antibody (a macrophage marker; ab6640, 1:200 dilution; Abcam, Cambridge, MA, United States) were used for immunohistochemical analysis of mouse tumors. In each case, at 400 × magnification, at least three fields at showing the highest density of positive macrophage infiltration were selected. CD68 and CD86 immunoreactivity was scored based on staining intensity (0, 1, and 2 indicating negative, weak, and strong staining, respectively) and the percentage of positive tumor cells per core (0, 1, 2, and 3 indicating < 6%, 6–25%, 25–50%, and > 50%, respectively). The number of macrophages stained in the field was determined. The percentage of positive cells was calculated as the number of positive cells/total cells × 100. The two scores were multiplied and the result was equal to or greater than 2, indicated positive expression.

### Cell Proliferation Assay

MDA-MB-231 cells were cultured under different conditions (CTRL, Co-CM1, Co-CM1 + FF, Con-CM, Con-CM + FF) and were added with 20% fresh medium for 12, 24, 36, 48, 60, and 72 h. The Cell Counting Kit-8 (CCK-8) assay was performed following the manufacturer’s protocols (HY-K0301, MCE, United States). Tumor cells were seeded in 96-well plates at a density of 5,000 cells/well. At each measurement point, the cells were incubated with complete medium containing 10% CCK-8 reagent for 2 h. Absorbance was measured at 450 nm.

### Human Breast Cancer Datasets and Tissue Specimen Preparation

Publicly available datasets with gene expression profiles of TNBC patient-derived tumor and normal tissues along with the related clinical information were obtained from the Gene Expression Omnibus (GEO) (Edgar et al., 2002). Twenty fresh TNBC tissue samples and paired normal breast tissue samples were obtained after surgery. The use of samples was approved by the Ethics Committee of Harbin Medical University Cancer Hospital and conformed to the provisions of the Declaration of Helsinki. All patients signed informed consent forms.

### Centrifugal Method for Collecting Interstitial Fluid

Centrifugation method was adopted to isolate interstitial fluids from tumors of human and mice. After anesthesia with pentobarbital (50 mg/kg), the body temperature of mice was maintained at 36.5–37.5°C. The anesthetized mice were immediately transferred to an incubator with a temperature of 20–24°C and 100% relative humidity. The tumor was excised, and the mice were sacrificed through cervical dislocation. The tumor tissue was flushed with saline to remove any blood from its surface, and excess saline was removed using filter paper. The tumor was transferred to a 2-mL centrifuge tube. The pre-weighed centrifuge tube was equipped with a nylon net basket with an aperture of 15 μm × 20 μm wherein the tumor was loaded. The tube was reweighed and centrifuged at 4°C. Tumors between 0.25 and 1.0 g were transferred intact to the tube. Larger tumors (>1 g) needed to be cut, then immediately brought back to the incubator. The basket containing the sample was transferred to another pre-weighed centrifuge tube, and the tumor fluid was collected. Samples were centrifuged at a speed of 100 rpm until they settled at the bottom of the tube.

### Glucose Assay

Supernatants from the co-cultured media of tumor cells and macrophages were centrifuged and collected at 0, 48, and 72 h. The 4T1 tumor tissues were excised, and the interstitial fluid was collected from mice 14 and 28 days after inoculation. The methods for interstitial fluid collection were the same as described previously. introduced earlier. The glucose concentration test was performed according to the manufacturer’s instructions for the glucose assay kit (A154-2-1, Nanjing Jiancheng). A 2.5 μL sample was mixed with 250 μL of reagent and incubated for 10 min at 37°C. The absorbance of the sample was read at a wavelength of 505 nm.

### Kyoto Encyclopedia of Genes and Genome Analysis and Gene Set Enrichment Analysis Analyses

Kyoto encyclopedia of genes and genome (KEGG) was used for pathway analysis. Gene count > 2 and *p* < 0.05 were set as the threshold. Gene set enrichment analysis (GSEA) was used to test the sets of related genes that might be systematically altered in TNBC. The details have been provided elsewhere (Subramanian et al., 2005).

### Small-Interfering RNA Transfection

For the small RNA interference assay, small-interfering RNA (siRNAs) were chemically synthesized by GenePharma Co., Ltd. (Shanghai, China). Macrophages were transfected using Lipofectamine^TM^ RNAiMAX Transfection Reagent (Thermo Fisher Scientific Inc.), and 2.5 × 10^4^ macrophage cells from the Co-CM1 group were planted on a 6-well plate. When the attached cells grew to 60–80%, 9 μL Lipofectamine^®^ RNAIMAX reagent was added to 150 μL Opti-Mem^®^ medium for dilution, 30 pmol siRNA was added to 150 μL Opti-Mem^®^ medium for dilution, and then 150 μL of each dilution was mixed. Next, 250 μL of the siRNA-liposome complex was added to each well. The cells were then incubated at 37°C for 2 days, and the transfected cells were analyzed. After successful transfection, cells were treated with FF.

### Mouse Model of Breast Carcinoma, Treatment Dose, and Measurements

Six-week-old female BALB/c nude mice were purchased from Beijing Vital River Laboratory Animal Technology. In brief, 1 × 10^5^ 4T1 cells were injected into the intramammary gland fat pad of the mice. Four groups (5–8 mice/group) were established 5 days after cell inoculation. Overall, 400 μg MIAP410 was injected into the mammary fat pad every other day, as described previously (Willingham et al., 2012). FF at a dose of 50 μg was injected intraperitoneally every 2 days. The body weight of each mouse was measured at 0, 2, 4, and 6 days after administration. The tumor volume was calculated using the formula V = ab2 × π/6, where a and b represent the length and width, respectively. Early-stage tumors were harvested 14 days after inoculation, whereas late-stage tumors were harvested 28 days after treatment. Except for the mice that were used to measure glucose concentration, other mice were sacrificed by cervical dislocation. Tumor weight was also measured. Tumor tissues were fixed in 10% formalin and embedded in paraffin for immunohistochemical analysis. Animal studies were approved by the Scientific and Ethical Committee of the Cancer Hospital/Institute of Harbin Medical University.

### Statistical Analysis

Quantitative data were represented as the mean ± SD. Statistical analysis was performed using SPSS 17.0 and GraphPad Prism 7.0. Student’s *t* test was performed to compare the two groups. Differences among the three groups were analyzed using one-way analysis of variance (ANOVA). Progression-free survival (PFS) curves were generated using the Kaplan–Meier method and analyzed using the log-rank test. Statistical significance was defined as *P* < 0.05. Differentially expressed genes (DEGs) were identified in the R software with packages “limma” and “sva.” The graphs were drawn with packages “ggrepel,” “ggplot2,” and “pheatmap.” CIBERSORT was performed in the R software with packages “preprocessCore,” “parallel,” “e1071,” and the source of “CIBERSORT.R.”

## Results

### The Function of Macrophages Was Impaired in the Breast Cancer TME

**FIGURE 1:**
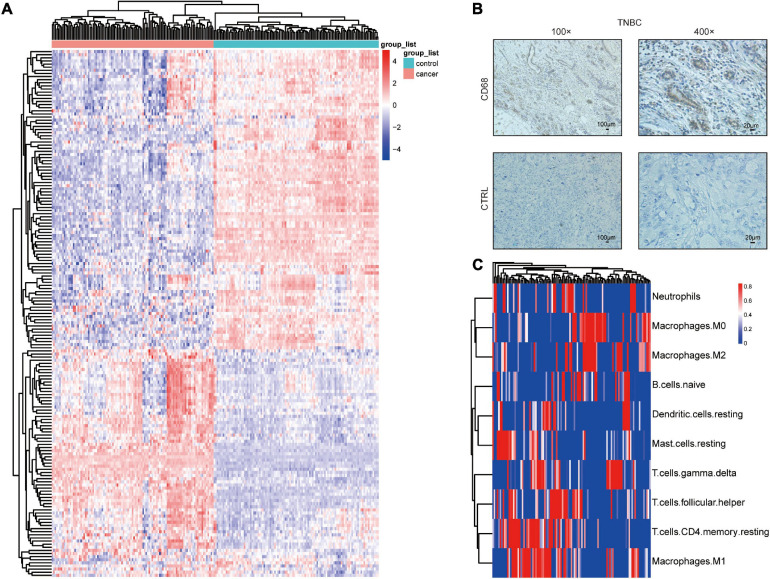
Macrophage distribution in TNBC. **(A)** Differential expression of immune-related genes in TNBC and normal tissues. **(B)** CD68 immunohistochemical staining in TNBC.**(C)** Heatmap showing the proportions of 22 immune cells predicted in TNBC.

**FIGURE 2:**
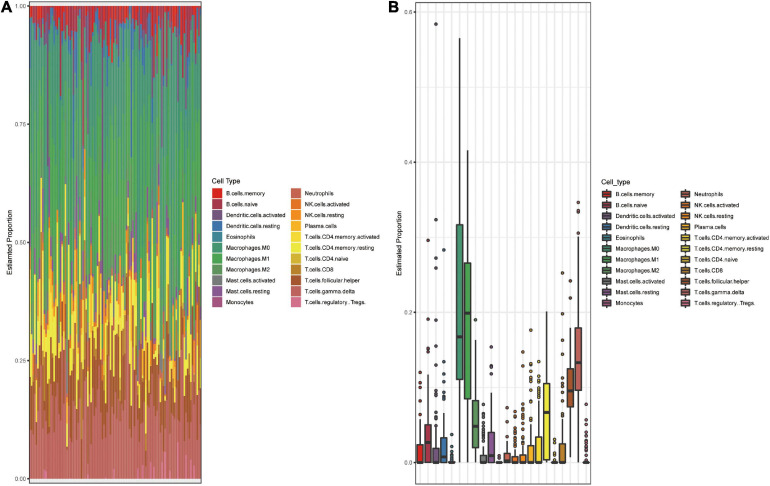
Landscape of immune cell subsets infiltration in TNBC. **(A)** Bar plot shows composition of 22 immune cell subsets in each TNBC sample (GSE58812). **(B)** Box plot shows the overall level of 22 immune cells proportion in GSE58812. The results were generated using the R software with the source “CIBERSORT.R.”

**FIGURE 3:**
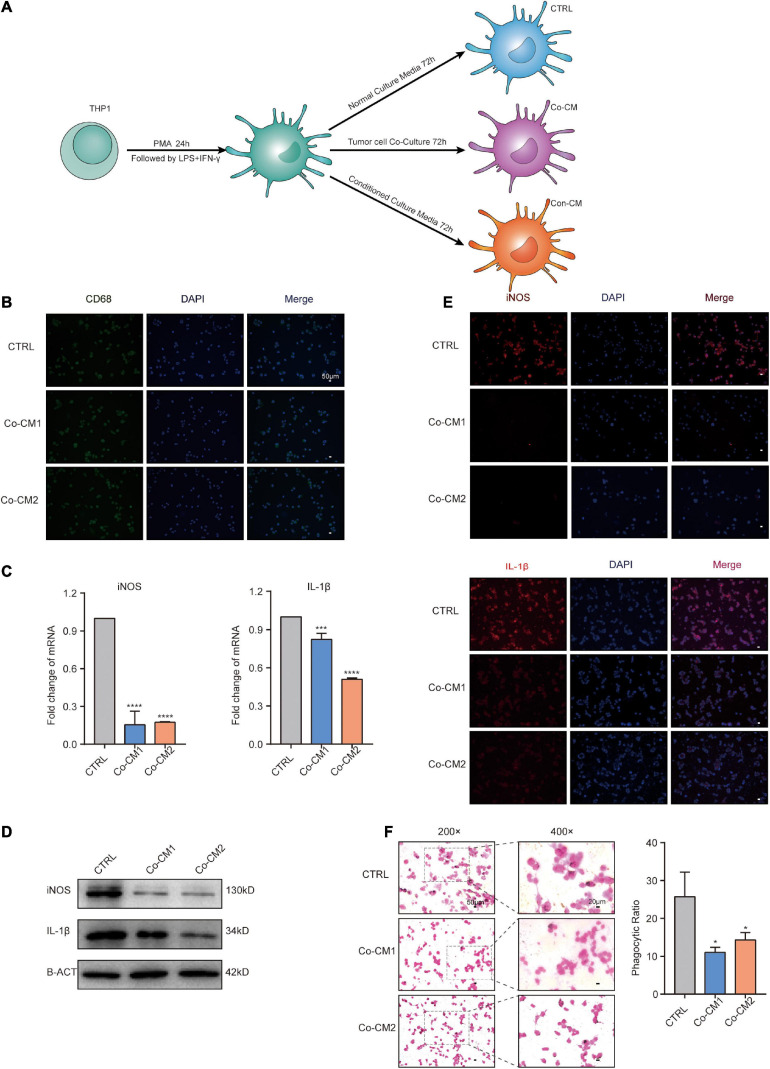
The function of macrophages was impaired in the TME. **(A)** Procedure for culture of macrophages under different culture conditions. **(B)** Immunofluorescence (IF) staining for CD68 in macrophages from CTRL, Co-CM1, and Co-CM2 groups counterstained with DAPI. **(C)** Real-time PCR analysis of iNOS and IL-1β expression in three kinds of macrophages (CTRL, Co-CM1, and Co-CM2). The results are shown as the mean ± SD. ***p<0.001 and ****p<0.0001. **(D)** iNOS and IL-1β protein expression levels are determined by western blotting. **(E)** Representative confocal immunofluorescence images of macrophages from CTRL, Co-CM1, and Con-CM groups stained for iNOS and IL-1β (red). **(F)** Eosin staining for macrophage phagocytosis of ink granules. The phagocytic ratio is calculated from 200 cells. *p<0.05.

Using the GEO datasets GSE58812 (Jezequel et al., 2015) and GSE112825 (Santucci-Pereira et al., 2019), the expression profiles of 247 immune-related genes from 107 TNBC and 109 normal breast tissue samples were compared and significant differences were observed (Figure 1A). Macrophages, which account for a large proportion of immune cells, may contribute more to this difference because of their location and type (Forssell et al., 2007; Algars et al., 2012). There was indeed an increase in the CD68 + macrophage population in breast cancer tissues (Figure 1B). CIBERSORT was used to estimate the fraction of 22 immune cell types in tumor tissues through a leukocyte gene signature matrix, termed LM22, which contains 547 genes that distinguish 22 human hematopoietic cell phenotypes (Figure 1C). T cells and macrophages are the two largest types, but the proportion of subtypes of these two types of cells varies greatly from individual to individual (Figure 2A). Overall, M1 and M0 macrophages accounted for a large proportion of triple-negative breast cancer tissues (Figure 2B). We further explored how the function of M1 macrophages was altered after they entered the TME. THP-1 cells were first induced by PMA and then exposed to LPS + IFN-γ. To mimic the TME, macrophages were then co-cultured with tumor cells that had been cultured to approximately 70% confluency for another 72 h (Figure 3A). These cells were all successfully induced into macrophages in both the control and CO-CM groups, expressing the common marker molecule CD68 of the macrophage lineage (Figure 3B). The function of M1 macrophages was further investigated. The mRNA and protein levels of IL-1β and iNOS were examined and immunofluorescence was performed. IL-1β and iNOS levels significantly reduced in co-cultures with tumor cells (Figures 3C–E) along with a decrease in phagocytosis (Figure 3F). However, it is not known whether the pro-tumor properties of M1 macrophages are enhanced in this microenvironment. IL-10, a marker of M2 macrophages, was also detected at the protein level, but no significant change was observed (Supplementary Figure 1A). These observations indicate that M1 macrophage function is impaired within the TME.

### Fatty Acid Catabolism and Glycolysis of Macrophages Decreased in the TME

**FIGURE 4:**
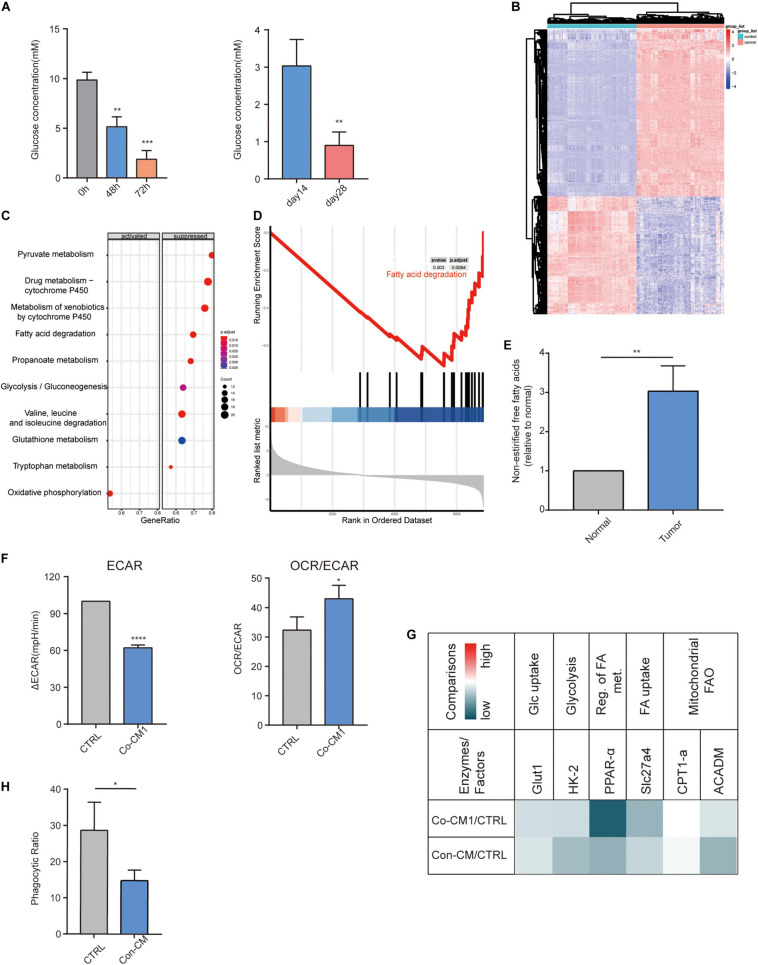
Fatty acid catabolism and glycolysis decreased in macrophages. **(A)** Glucose concentration in the co-culture medium of MAD-MB-231 cells and macrophages at 48 h and 72 h as well as in the interstitial fluid from early-stage (day 14) and late-stage (day 28) 4T1 tumors (*n* = 3). **p < 0.01, ***p < 0.001. **(B)** Heatmap of 1,836 differentially expressed genes between TNBC and normal tissues. **(C)** Significant enrichment activated and inhibited KEGG pathway. The vertical term is the name of the KEGG term, and the length of the horizontal plot represents the gene proportion. The color represents the *p* value. The area of the circle represents the gene count. **(D)** GSEA-based KEGG-enrichment plots of representative gene sets from suppressed pathway: Fatty acid degradation. **(E)** The relative ratio of the concentration of non-esterified free fatty acids in human TNBC and normal breast tissues. **p < 0.01. **(F)** ECAR and OCR to ECAR ratio in CTRL and Co-CM1 groups. *p < 0.05, ****p < 0.0001. **(G)** Relative transcription levels of glycolysis- and fatty acid catabolism-related factors and enzymes: Co-CM1 vs. CTRL and Con-CM vs. CTRL. **(H)** Relative phagocytic ratio in macrophages from Con-CM and CTRL groups. *p < 0.05.

M1 macrophages are damaged following their entry into the TME. We speculate that the metabolism of macrophages may have been altered within the TME. We measured the glucose concentration in the medium from co-cultured tumor cells and macrophages at 0, 48 and 72 h, as well as in the interstitial fluid of early (14 days) and late (28 days) breast tumors from mice. Glucose levels decreased in the medium with the processing of cultures as well as in the interstitial fluid of the advanced tumors (Figure 4A). The heatmap showed 1,836 differentially expressed genes between the tumor and normal tissues (Figure 4B). We performed KEGG and GSEA on co-expressed genes to explore the potential activation and suppression of metabolic pathways in the tumor. Our results revealed the suppression of the FA degradation pathway (Figures 4C,D). Thus, the TME is abundant in FAs. We then measured the non-esterified free fatty acids concentration in the interstitial fluid of human breast cancer tissues and normal tissues, and found that non-esterified free fatty acids levels were higher in the tumor tissue (Figure 4E). We further explored the changes in macrophage metabolism in such environments and found that ECAR, a measure of glycolysis, decreased and the OCR/ECAR ratio increased in macrophages from the co-culture system (Figure 4F). To study the metabolic pathways of macrophages within the TME, we incubated them in a low-glucose-conditioned medium supplemented with palmitic acid and oleic acid to mimic the tumor metabolic microenvironment. The transcripts of factors that participate in glycolysis and FA catabolism were all reduced (Figure 4G). IL-1β and iNOS levels also decreased in the conditioned media (Figure 3E), as well as phagocytic activity (Figure 4H). The above results demonstrate the downregulation of glycolysis and FA catabolism of macrophages within the TME.

### Enhancing Macrophage FA Catabolism Within the Breast TME Can Reprogram Them and Restore Their Functions

**FIGURE 5:**
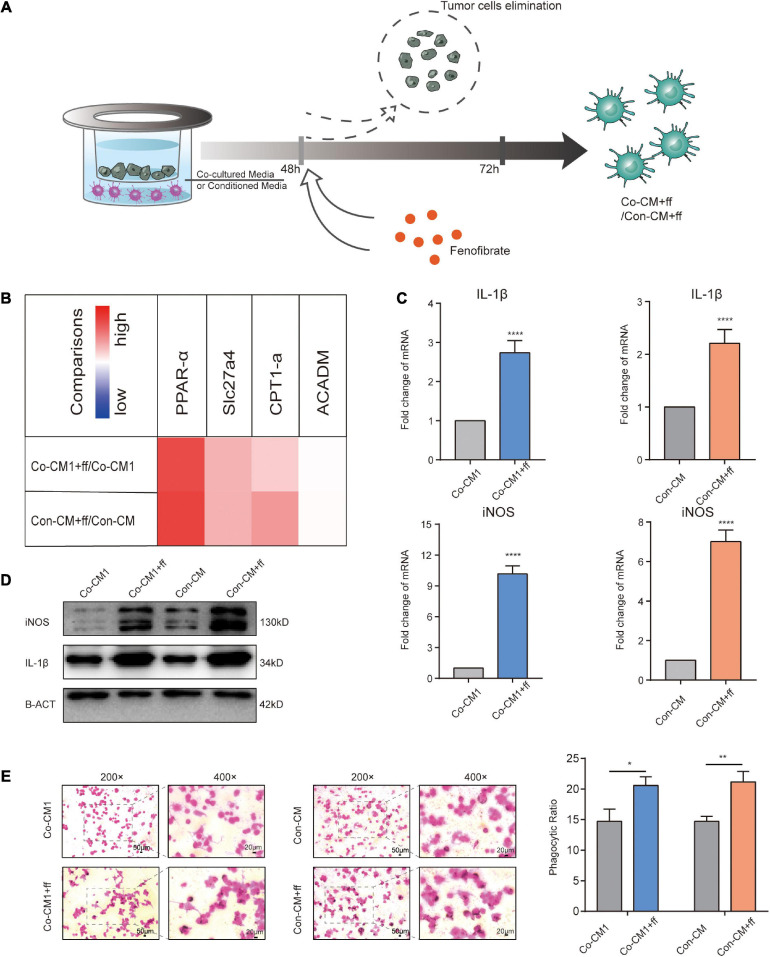
Promoting FA catabolism reprogrammed macrophages to resume their functions. **(A)** Description of the intervention process in Co-CM and Con-CM groups by FF. **(B)** Transcript levels of related factors and enzymes involved in FA catabolism in Co-CM1 or Con-CM groups treated with FF vs. Co-CM1 and Con-CM group, respectively (*n* = 3). **(C)** Transcription of iNOS and IL-1β in Co-CM1 and Con-CM groups treated with FF. ****p < 0.0001. **(D)** Western blot analysis of iNOS and IL-1β expression in Co-CM1, Con-CM, Co-CM1 + FF, and Con-CM + FF groups. **(E)** Phagocytosis of ink granules in Co-CM1, Con-CM, Co-CM1 + FF, and Con-CM + FF groups, as measured from the phagocytic ratio. *p < 0.05, **p < 0.01.

**FIGURE 6:**
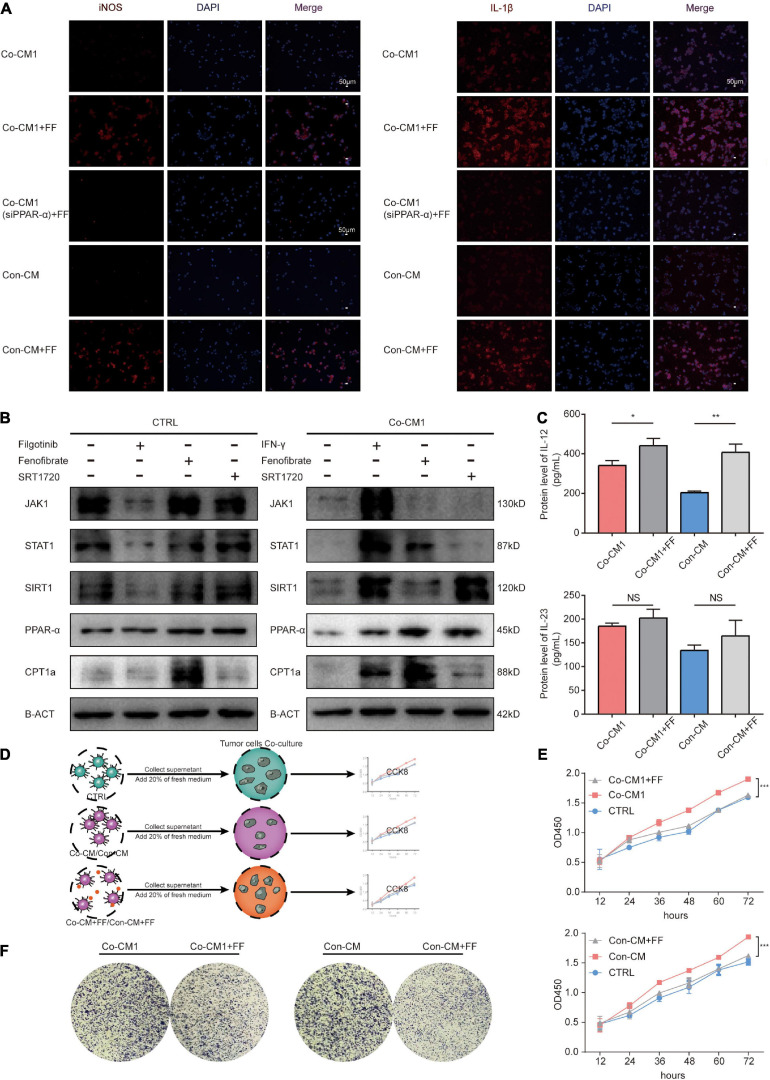
Fenofibrate re-established macrophages to enhance their antitumor function by activating the PPAR-α-STAT1 and FAO positive feedback loop pathways. **(A)** Immunofluorescence staining for the expression of cytokines IL-1β and iNOS in Co-CM1, Con-CM with and without FF treatment, as well as Co-CM1 (siPPAR-α) + FF groups. **(B)** SIRT1, JAK1-STAT1, and FAO pathways were assessed by western blot analysis in CTRL and Co-CM1 groups. **(C)** ELISA of the IL-12 and IL-23 secretion by macrophages in Co-CM1 and, Con-CM with and without FF treatment. *p < 0.05, **p < 0.01. **(D)** Elucidation of tumor cells proliferation from culture medium of different conditioned macrophages. **(E)** Proliferation of MDA-MB-231 cells treated with conditioned media from different macrophages. ***p < 0.001. **(F)** Clone formation of MDA-MB-231 cells cultured in supernatant from different macrophages.

Given the large abundance of FAs in malignant tumor tissues (Kleinfeld and Okada, 2005; Halczy-Kowalik et al., 2019), in light with our results, we investigated whether re-activation of FA catabolism restores the normal function of M1 macrophages within the TME. After macrophages were co-cultured with tumor cells or in mimic-conditioned medium for 48 h, they were exposed to a PPAR-α agonist, fenofibrate, that increased FA catabolism for 24 h (Figure 5A). Transcriptional levels of FA catabolism-related factors increased in FF-treated macrophages (Figure 5B), and IL-1β and iNOS expression was significantly upregulated at the mRNA and protein levels, consistent with the results of the immunofluorescence assay (Figures 5C,D, 6A). The ability of macrophages to engulf ink was also improved (Figure 5E). Thus, we conclude that the increased catabolism of FAs in glucose-starved macrophages could re-establish them to regain their function.

### Enhancement of FA Catabolism in Macrophages Restored Their Function Through the PPAR-α–STAT1 Positive Feedback Loop and Fatty Acid Oxidation Pathways Within the Hypoglycemic Breast Cancer TME

We have shown that enhancement of FA catabolism could re-establish M1 macrophages to resume their function within the limited glucose TME. Fatty acid oxidation (FAO) can provide ATP to cells for survival, and studies have shown that ATP enhances the functions of macrophages (Wang et al., 2018). The JAK1-STAT1 signaling pathway is a classical pathway that regulates macrophage function (Gan et al., 2017; Xue et al., 2017). However, the potential targets of PPAR-α, including STAT1(McMullen et al., 2014), and the underlying mechanisms of action are not well understood. We investigated JAK1 and STAT1 protein expression levels in macrophages from CTRL and Co-CM1 groups and found that their expression was inhibited in macrophages co-cultured with tumor cells. FF-treated macrophages showed upregulated expression of FA catabolism-related factors, PPAR-α and CPT1A, as well as STAT1 in Co-CM1 cells (Figure 6B). The expression of STAT1 failed to change after treatment of macrophages from the CTRL group with FF. Silent information regulator transcript-1 (SIRT1) is reported to regulate various physiological processes such as cell cycle, metabolism, and inflammation. It has been reported that STAT1 promotes PPAR-α expression through SIRT1 (Kauppinen et al., 2013; Yoo et al., 2014). Therefore, we explored whether if a positive feedback loop exists between PPAR-α and STAT1 through SIRT1. Filgotinib (JAK1-STAT1 inhibitor, 1 μM) abrogated SIRT1 expression in the CTRL group, while IFN-γ (JAK1-STAT1 agonist, 10 μg/mL) promoted SIRT1 expression in the Co-CM1 group (Figure 6B). SIRT1 expression was upregulated by the JAK1-STAT1 pathway. We also treated macrophages with SRT1720 (SIRT1 agonist, 1 μM) and observed upregulation in the expression of PPAR-α and CPT1A (Figure 6B). In addition, no significant change in the nuclear factor kappa B (NFκB) signaling pathway was observed following treatment of macrophages with FF in this condition (Supplementary Figure 1B). To further confirm that FF can regulate the function of macrophages through PPARα, the Co-CM1 group was transfected with si-PPAR-α (Supplementary Figure 1C). The knockdown of PPAR-α expression led to the elimination of the recovery of IL-1β and iNOS expression in Co-CM1 group treated with FF (Figure 6A), thus confirming that FF upregulated the function of macrophages. These results suggest that the enhancement of FA catabolism by FF could reprogram macrophage function through the PPAR-α-STAT1 positive feedback loop and FAO in the limited glucose TME.

### Enhancing FA Catabolism Combined With Anti-CD47 Treatment Inhibited Tumor Proliferation *in vitro* and *in vivo*

**FIGURE 7:**
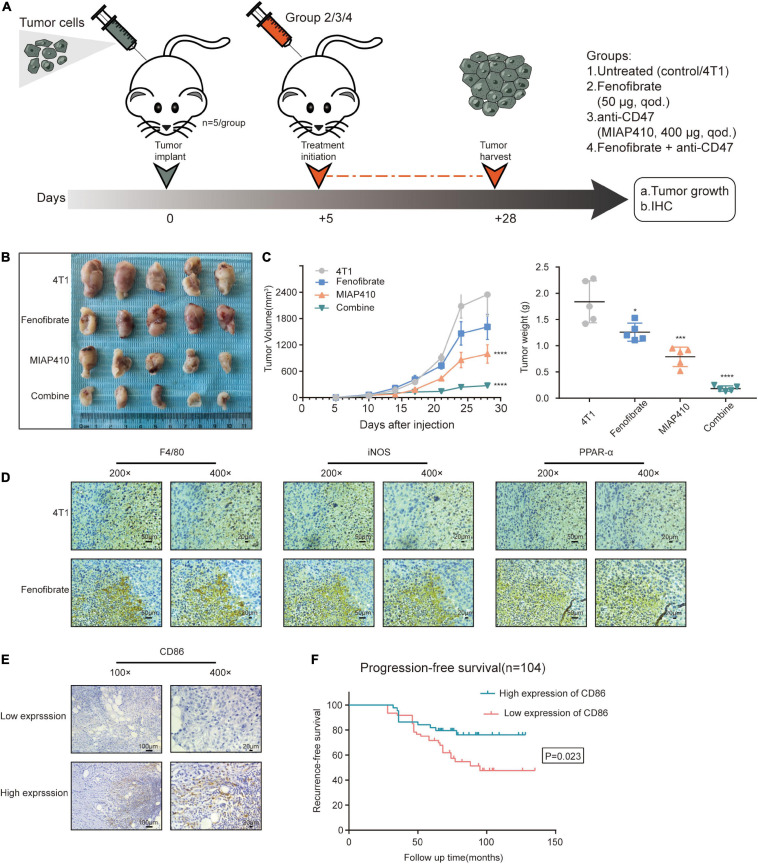
Fenofibrate combined with anti-CD47 inhibited tumor cell proliferation *in vivo*. **(A)** Experimental set up: Drug treatment model of tumor-bearing mice. **(B)** Images of tumors subjected to different treatments. **(C)** Tumor volume is determined by caliper measurements and is presented as mean (mm^3^) ± SEM. Tumor weights at day 28 are shown as mean ± SEM. *n* = 5/group. *p < 0.05, ***p < 0.001, ****p < 0.0001. **(D)** Immunohistochemical assay to confirm the expression of F4/80, iNOS, and PPAR-α in serial sections of tumor samples from FF and control groups. **(E)** Representative images of immunohistochemical staining for CD86 from human TNBC samples. **(F)** Kaplan-Meier recurrence-free survival curves of TNBC patients with low (*n* = 60) and high (*n* = 44) CD86 expression.

The function of M1 macrophages was reprogrammed, and their effects on tumor cells were further studied. The protein levels of IL-12 and IL-23 in the supernatant from the FF-treated macrophages were detected *via* ELISA. As expected, enhanced production of IL-12 was observed (Figure 6C), and the amount of IL-23 increased, but there were no statistically differences in these changes. The tumor cells were incubated with the supernatant from macrophages under different conditions. The cell proliferation assay showed that the proliferation of tumor cells was significantly inhibited in the Co-CM1 + FF and Con-CM + FF groups (Figures 6D,E). The colony formation of tumor cells also decreased (Figure 6F). Anti-CD47 therapy enhances phagocytosis of tumor cells by macrophages (Chao et al., 2012; Zhang et al., 2016). Therefore we evaluated whether FF and anti-CD47 therapy could exert synergistic antitumor effects. *In vitro*, 4T1 cells were treated with 10 μM FF for 48 h, and the cell viability was approximately 85.57 ± 17.96% (Supplementary Figure 1D). The 4T1 tumor-bearing BALB/c nude mice were treated with different drug interventions (Figure 7A). These mice showed regular activity and sleep rhythms, with good physical and mental functioning. There were no significant changes in diet or water intake, and no obvious adverse reactions were observed. In the short term (from 1 to 6 days after drug treatment), there was no significant difference in weight gain between the drug-treated and the control groups (Supplementary Figure 1E). At the end of the experiment, the tumor weights and volumes in mice treated with FF or FF combined with anti-CD47 were lower than those in the control mice (Figures 7B,C). Immunohistochemical staining for F4/80, iNOS and PPAR-α was performed with successive slides of mouse tumor tissue. F4/80 is as a proinflammatory macrophage cell marker in mice (Lee et al., 2018). The expression of PPAR-α in proinflammatory macrophages increased and macrophage function (increased expression of F4/80 and iNOS) was restored after FF administration in tumor-bearing mice (Figure 7D). To determine the relationship between antitumor macrophages and patient prognosis, we selected 104 TNBC patients who had undergone surgical treatment. Their tumor tissues were stained for CD86, which is often used as a marker for human proinflammatory macrophages (Dong et al., 2016; Matsui et al., 2017). The progression-free survival (PFS) curve showed that patients with high CD86 expression had better PFS (Figures 7E,F). These results suggest that antitumor macrophages play a crucial role in breast cancer progression. Taken together, tumor cell proliferation was inhibited in the tumor nutrient-challenged microenvironment by re-established macrophages following enhancement of the catabolism of FAs *in vitro* and *in vivo*.

## Discussion

This study provides a novel metabolic insight into macrophage-mediated progression of TNBC. We showed that the phagocytic function of macrophages was impaired within the TNBC microenvironment or in co-cultures with TNBC cell lines. This change was accompanied with a gradual decline in the glycolysis and FA catabolism of macrophages. Enhancing FA catabolism through treatment with FF, a key mediator of metabolic intervention, could restore the phagocytic functions of macrophages by activating the STAT1 signaling pathway and increasing in ATP production by FAO.

We also showed that FF alone or in combination with a CD47 antibody (MIAP410) could increase the ability of macrophages to inhibit tumor proliferation *in vivo* and *in vitro*. These findings reveal the mechanism underlying the metabolic reprogramming-driven functions of macrophages in the TME of TNBC and provide insights for novel therapeutic strategies for TNBC.

Components in the TME, particularly tumor-associated macrophages (TAMs), represent a hallmark of cancer that contributes to cancer progression. We have previously confirmed that TAMs in the TME of postmenopausal breast cancer patients are predominantly polarized toward an anti-inflammatory macrophage phenotype (Liu et al., 2017) and are associated with drug resistance and reduced survival (Ji et al., 2016; Li et al., 2020). In addition to anti-inflammatory macrophages TAMs include pro-inflammatory macrophages. The ability of M1 macrophages to engage in phagocytosis and production of proinflammatory cytokines largely relies on enhanced glycolysis (Mehta et al., 2017). However, increased glucose consumption by TNBC cells results in glucose deprivation within the TME. Thereby, it is difficult for macrophages to maintain their functions within the glucose-limited TME. Inhibition of glycolysis can reduce nitric oxide production and the phagocytic function of macrophages (Liu et al., 2018), which is consistent with our experimental results. Our results showed that the free FAs increase in the TME and that the FA catabolism is restrained in macrophages. Therefore, an intervention that switches glycolysis to FA catabolism may induce novel metabolic pathways to support macrophage reactivation and re-elicitation. PPAR-α is a central mediator of lipid metabolism that regulates FAO and promotes metabolic responses. Research has shown that the activation of PPAR-α promotes lipid catabolism and fatty acid oxidation in macrophages during mycobacterium infection, as well as phagosome maturation and antimicrobial response (Kim et al., 2017). This seems to differ from our pro-inflammatory results. Our results showed that fenofibrate, an agonist for PPAR-α activation, activated FA catabolism of macrophages in the glucose-limited and FA-rich TME, enhanced the expression of functional cytokines IL-1β and iNOS, and promoted the recovery of antitumor functions. Currently, it is widely believed that the JAK1/STAT1 signaling pathway is one of the major pathways of M1 macrophage activation (Gough et al., 2008). When this pathway is deregulated, phagocytosis of macrophages is inhibited (Lei et al., 2019). Indeed, we identified the involvement of the JAK1-STAT1 pathway in the above process. A study using database analysis predicted that STAT1 may be a potential target downstream of PPAR-α, possibly requiring activation by intermediate protein kinases (McMullen et al., 2014). PPAR-α has also been shown to activate STAT1 indirectly (Qin et al., 2012; Read et al., 2015; Yao et al., 2020). Previous studies have focused on the function of PPAR-α in macrophages under physical conditions. Macrophages mainly rely on glycolysis for energy supply, and PPAR-α does not play a critical role in metabolism. However, our study was conducted on the resting state of complete polarization of M1 macrophages under a glucose-limited microenvironment, which may provide a prerequisite for STAT1 activation. Furthermore, activation of the STAT1 pathway promotes the positive feedback loop of PPAR-α by upregulating SIRT1 expression.

**FIGURE 8:**
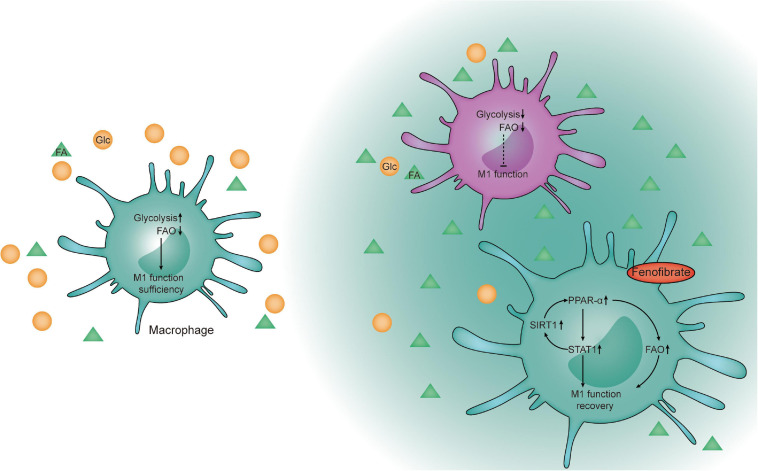
Schematic diagram. Proposed model of fenofibrate-mediated activation of the FAO pathway to re- establish macrophages to recover their functions through the FAO and PPAR-α-STAT1 positive feedback loop pathways within the glucose-limited TME.

Cancer immunotherapies have been extended to innate immune checkpoints, especially macrophage checkpoint blockade, which interferes with the detection and clearance of malignant cells through phagocytosis. Although the “don’t eat me” signal CD47–SIRPα axis in cancers is crucial for escape from macrophage-mediated immune recognition and phagocytic clearance, other mechanisms may also contribute to the antitumor effects associated with increased activity in and recruitment of macrophages to tumor tissue (Xiao et al., 2015). In our study, the phagocytic function of macrophages was impaired in the TME and it could be restored by the catabolism of FAs *in vitro* and *in vivo*. FF combined with anti-CD47 significantly inhibited tumor cell proliferation in tumor-bearing mice. Cancer cells can be recognized through anti-CD47 therapy, and cancer clearance function can be increased through metabolic reprogramming. A limitation is that the THP1 cell line was used in the experiments. The manipulation of monocytes from human peripheral blood to induce macrophages may be closer to that observed in the human physical environment. However, at present, there is a lack of ideal isolation methods. The potential of differentiation is poor and a limited number of cells can be obtained. So further exploration is still needed. In the study, our research focus is mainly on restoring the anti-tumor and phagocytic function of macrophages by regulating lipid metabolism in a glucose-competitive tumor microenvironment. Other immunosuppressive factors including lactic acid and hypoxia et al, were not considered dominant influencing factors. In the future, we will conduct research to explore the interaction effect on the macrophages when various factors in the tumor metabolic microenvironment are considered together. In conclusion, our results indicate that enhancing FA catabolism of macrophages in the TME can re-establish their antitumor functions through FAO and PPAR-α–STAT1 positive feedback loop pathways (Figure 8). Combination of FF and anti-CD47 treatment may be a potential therapy for patients with TNBC.

## Data Availability Statement

The datasets presented in this study can be found in online repositories. The names of the repository/repositories and accession number(s) can be found in the article/Supplementary Material.

## Ethics Statement

The studies involving human participants were reviewed and approved by the Ethics Committee of Harbin Medical University Cancer Hospital. The patients/participants provided their written informed consent to participate in this study. The animal study was reviewed and approved by the Scientific and Ethical Committee of the Cancer Hospital/Institute of Harbin Medical University.

## Author Contributions

QZ and HJ raised the concept, designed the study, and supervised the project. YG, XN, and LY carried out most of the experimental work. XY collected samples and patients information. YY conducted animal experiments. YW analyzed the data. HJ and YG wrote the manuscript. All authors contributed to the article and approved the submitted version.

## Conflict of Interest

The authors declare that the research was conducted in the absence of any commercial or financial relationships that could be construed as a potential conflict of interest.

## Supplementary Material

Supplementary Figure 1 **(A)** Protein expression level of IL-10 in Co-CM1 and CTRL groups. **(B)** Western blot analysis of NFκB p65 expression in Co-CM1 and Co-CM1 + FF groups. **(C)** Protein level of PPAR-α after siRNA treatment in the Co-CM1 group. **(D)** The histogram shows the cell viability of 4T1 cells treated with fenofibrate (10 μM) *in vitro*. **(E)** The short-term safety of drug administration was assessed by changes in body weight on days 2, 4, and 6 of the week.
