# Supplementary figures and images for: Enhancing Fatty Acid Catabolism of Macrophages Within Aberrant Breast Cancer Tumor Microenvironment Can Re-establish Antitumor Function
Source: Front Cell Dev Biol. 2021 Apr 15;9:665869. doi: 10.3389/fcell.2021.665869 (PMC8081981; doi:10.3389/fcell.2021.665869)

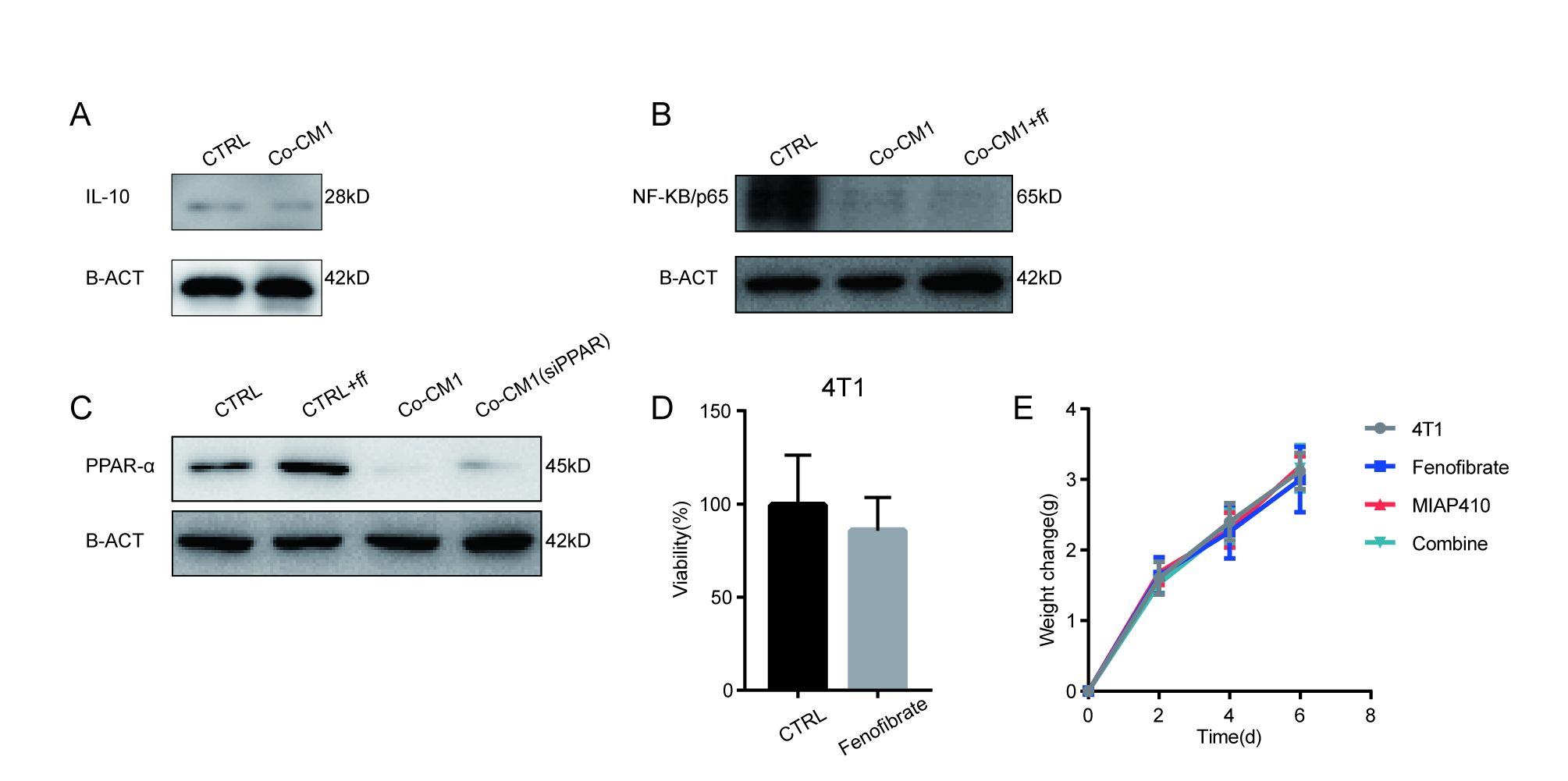

Supplement: Supplementary Figure 1 — (A) Protein expression level of IL-10 in Co-CM1 and CTRL groups. (B) Western blot analysis of NFκB p65 expression in Co-CM1 and Co-CM1 + FF groups. (C) Protein level of PPAR-α after siRNA treatment in the Co-CM1 group. (D) The histogram shows the cell viability of 4T1 cells treated with fenofibrate (10 μM) in vitro. (E) The short-term safety of drug administration was assessed by changes in body weight on days 2, 4, and 6 of the week. [file Image_1.TIF]
